# Machine learning classifier to identify clinical and radiological features relevant to disability progression in multiple sclerosis

**DOI:** 10.1007/s00415-021-10605-7

**Published:** 2021-05-10

**Authors:** Silvia Tommasin, Sirio Cocozza, Alessandro Taloni, Costanza Giannì, Nikolaos Petsas, Giuseppe Pontillo, Maria Petracca, Serena Ruggieri, Laura De Giglio, Carlo Pozzilli, Arturo Brunetti, Patrizia Pantano

**Affiliations:** 1 Department of Human Neurosciences, Sapienza University of Rome, Viale dell’Università, 30, 00185 Rome, Italy; 2 Dipartimento di Scienze Biomediche Avanzate, Università degli Studi di Napoli Federico II, Naples, Italy; 3 Institute for Complex Systems, Italian National Research Council, Rome, Italy; 4 Department of Radiology, IRCCS NEUROMED, Pozzilli, Italy; 5 Dipartimento di Ingegneria Elettrica e delle Tecnologie dell’Informazione, Università degli Studi di Napoli Federico II, Naples, Italy; 6 Dipartimento di Neuroscienze, Scienze Riproduttive e Odontostomatologiche, Università degli Studi di Napoli Federico II, Naples, Italy; 7 Neuroimmunology Unit, IRCSS Fondazione Santa Lucia, Rome, Italy; 8 Neurology Unit, Medicine Department, San Filippo Neri Hospital, Rome, Italy

**Keywords:** Multiple sclerosis, Machine learning, Magnetic resonance imaging, Disability progression

## Abstract

**Objectives:**

To evaluate the accuracy of a data-driven approach, such as machine learning classification, in predicting disability progression in MS.

**Methods:**

We analyzed structural brain images of 163 subjects diagnosed with MS acquired at two different sites. Participants were followed up for 2–6 years, with disability progression defined according to the expanded disability status scale (EDSS) increment at follow-up. T2-weighted lesion load (T2LL), thalamic and cerebellar gray matter (GM) volumes, fractional anisotropy of the normal appearing white matter were calculated at baseline and included in supervised machine learning classifiers. Age, sex, phenotype, EDSS at baseline, therapy and time to follow-up period were also included. Classes were labeled as stable or progressed disability. Participants were randomly chosen from both sites to build a sample including 50% patients showing disability progression and 50% patients being stable. One-thousand machine learning classifiers were applied to the resulting sample, and after testing for overfitting, classifier confusion matrix, relative metrics and feature importance were evaluated.

**Results:**

At follow-up, 36% of participants showed disability progression. The classifier with the highest resulting metrics had accuracy of 0.79, area under the true positive versus false positive rates curve of 0.81, sensitivity of 0.90 and specificity of 0.71. T2LL, thalamic volume, disability at baseline and administered therapy were identified as important features in predicting disability progression. Classifiers built on radiological features had higher accuracy than those built on clinical features.

**Conclusions:**

Disability progression in MS may be predicted via machine learning classifiers, mostly evaluating neuroradiological features.

**Supplementary Information:**

The online version contains supplementary material available at 10.1007/s00415-021-10605-7.

## Introduction

Clinical progression and disability accumulation are highly heterogeneous in all phenotypes of multiple sclerosis (MS) [1]. The variability of the disease course is guided by inflammation, axonal degeneration and remyelination phenomena [2], but how these phenomena influence the course of the disease is not yet clear. Several disease modifying treatments are now available to improve long-term prognosis of patients with MS [3]. However, choosing the right treatment is still a challenge for clinicians who need to balance the drug safety profile with the risk of disability progression on individual basis.

Neuroimaging techniques are powerful tools to investigate MS [4] and Magnetic Resonance Imaging (MRI) findings are considered good predictors of conversion from clinically isolated syndrome (CIS) to clinically definite MS [5], as well as of long-term disability [6, 7]. Several MRI studies have highlighted various aspects of tissue damage in MS [8] demonstrating a prognostic role of T2-hyperintense lesions, global and cortical atrophy [9], as well as that of damage to some key structures, such as thalamus [10] and cerebellum [11, 12]. Among these, lesion burden appeared to be a relevant predictor of long-term cognitive outcome [13] and disease progression [14]. Further factors anticipating disability progression in MS have been shown to be structural and microstructural damage in the cerebellum [15], thalamus [16] and normal appearing white matter (NAWM) [17].

Recently, machine learning (ML) techniques have been applied to analyze clinical and radiological data in MS. Indeed, ML classifications represent a valuable tool for predicting conversion from CIS to MS [18] and to reliably distinguish patients with MS from healthy subjects [19]. Classifiers were also able to identify brain regions, and both functional and structural connections relevant to better understand the disease [19].

Till now, ML techniques have been applied mainly for diagnostic purpose and the utility of those tools for the prediction of disability progression has not been explored yet. This point is crucial in MS management, considering that identifying patients with higher risk of disability progression might promptly recognize subjects who may benefit from a more aggressive therapeutic approach.

We hypothesized that a data-driven approach on clinical and MRI data may predict disability progression in single subjects with MS. We tested this hypothesis in this pilot study, applying ML classifiers built on clinical data and neuroradiological features. Moreover, we investigated among clinical and neuroradiological features what ML classifiers are able to identify the most important factors in predicting disability progression in MS.

## Materials and methods

**Fig. 1:**
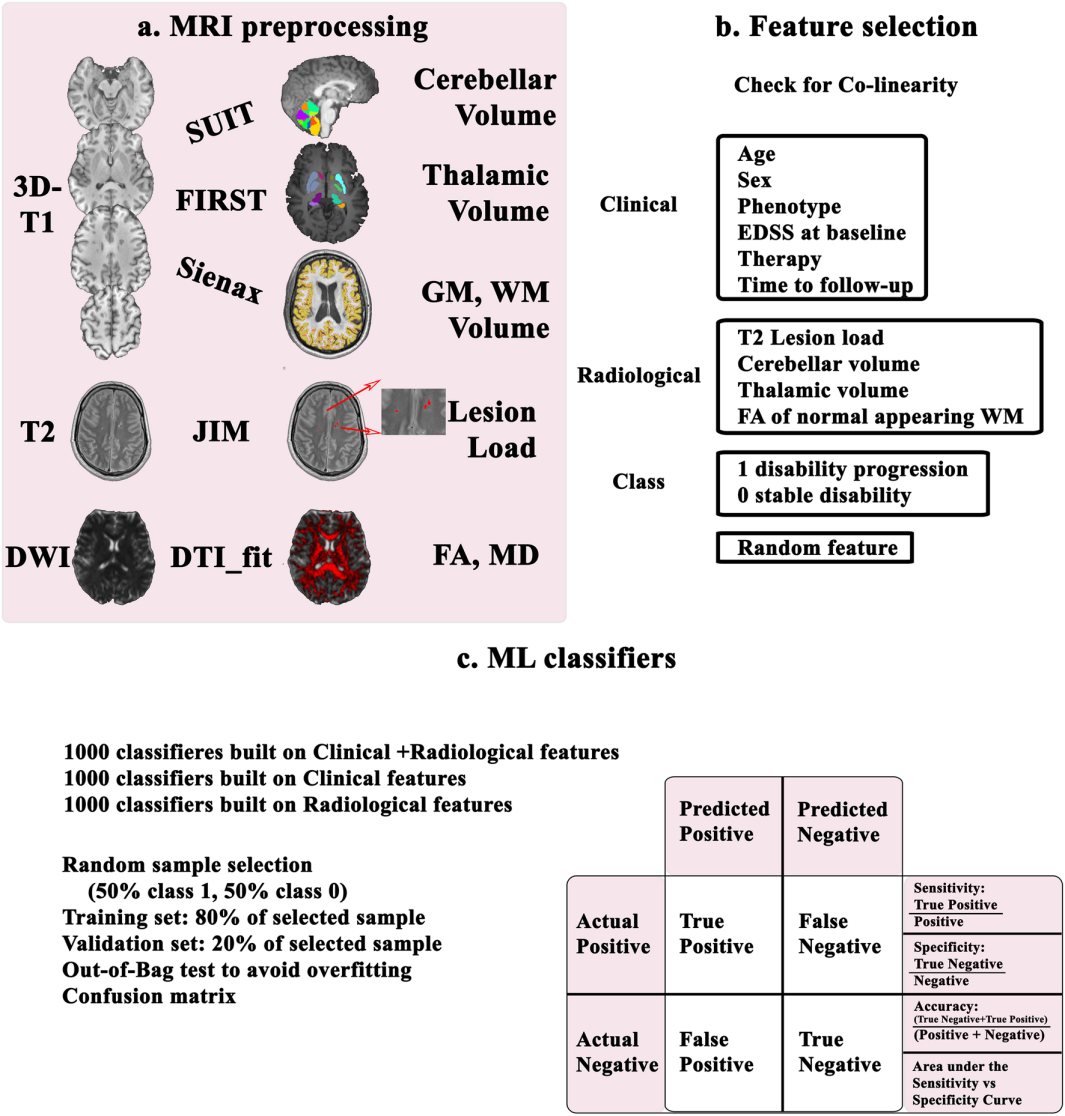
Flowchart of the methods. **a** MRI preprocessing. From 3D-T1-weighted images cerebellar volume was calculated via SUIT, thalamic volume using FSL’s FIRST and gray matter and white matter volumes via FSL’s SIENAX. On the T2-weighted images lesions were identified and segmented using JIM, to calculate the lesion load. By combining lesion and white matter masks, we calculated the normal appearing white matter mask for each subject. From diffusion-weighted images both fractional anisotropy and mean diffusivity maps were calculated, and results and were combined with the normal appearing mask previously obtained to extract microstructural metrics of the normal appearing white matter. **b** Feature selection. Clinical and neuroradiological, were selected together with binary classes (stable patients = 0, patients with disease progression = 1) and a random feature and used to describe the sample of patients with multiple sclerosis. **c** Machine learning classifier. After having checked features for co-linearity, a random forest classifier was applied 1000 times feature built on both clinical and radiological features, clinical features alone, radiological features alone. Out-of-Bag test was used to avoid overfitting and performances were evaluated via the confusion matrix of the surviving classifiers. *DWI* diffusion-weighted images, *GM* gray matter, *WM* white matter, *FA* fractional anisotropy, *MD* mean diffusivity

A flowchart showing the methods of this work is proposed in Fig. 1. A detailed step-by-step description of all the procedures follows.

### Sample selection

Study protocols were approved by the ethical committee of Policlinico Umberto I/Sapienza University (Rome, Italy, Site 1) and Ethics Committee for Biomedical Activities “Carlo Romano” of Federico II University (Naples, Italy, Site 2) as appropriate. All subjects provided written informed consent.

We analyzed clinical and MRI data of subjects with MS collected by two centers: the Human Neuroscience Department of Sapienza University (Site 1) and the MS center of the Federico II University (Site 2).

From both sites, clinical evaluations were performed between 2010 and 2018, while all MRI acquisitions were performed between 2010 and 2015. MS patients were included according to the following selection criteria: diagnosis of MS according to the Mc Donald’s criteria [20, 21]; age between 18 and 70 years; clinical assessment and MRI examination not more than one month apart; clinical follow-up available after a minimum of 2 years from the MRI examination. On both visits, patients were examined by expert neurologists assessing the clinical status via the Expanded Disability Status Scale (EDSS). At the follow-up examination, disability progression was defined as 1.5-point increase for patients with a baseline EDSS of 0, 1 point for scores from 1.0 to 5.0, and 0.5 points for scores equal or higher than 5.5 [22].

Finally, at both sites patients underwent a MRI scan with a 3 T system (Verio, Siemens equipped with a 12-channelhead coil at Site 1; Trio, Siemens, equipped with a 8-channel head coil at Site 2), including tridimensional (3D) T1-weighted (-w); T2-w, either dual echo or FLAIR; diffusion-w images. Detailed information on acquisition parameters are reported in Supplementary Materials.

### MRI data analysis

T2-weighted lesion load (T2LL) was calculated independently by three expert operators, with five to eleven years of experience, who identified the hyperintense WM lesions, using a semi-automatic technique (Jim, Xinapse System, Leicester, UK; http://www.xinapse.com). In the two sites, a lesion mask common to all patients was built including brain areas presenting lesions in at least the 10% of the sample [23].

Gray (GM) and white (WM) matter volumes were calculated via SIENAX, implemented in FSL environment (https://fsl.fmrib.ox.ac.uk/fsl/fslwiki/), while thalamic volumes (calculated as the sum of right and left) were obtained via FSL FIRST. Cerebellar lobules, summed to obtain total cerebellar volume, were identified and calculated using the Spatially Unbiased Infratentorial Toolbox (SUIT, http://www.diedrichsenlab.org/imaging/suit.htm), implemented in SPM12. Lastly, GM, WM, thalamic and cerebellar volumes were normalized to the scaling factor (obtained in SIENAX) to account for head size.

Diffusion maps were generated using the DTI_fit model, part of the FSL Diffusion Toolbox (http://www.fmrib.ox.ac.uk/fsl/fdt). Combining the results of FA, or MD, for each patient with WM mask and the image of the common lesions, we obtained individual maps of the FA, or MD, for NAWM, that were further averaged to obtain a single value for FA-NAWM and MD-NAWM [23].

### Statistical analyses

Statistical analyses were performed with Matlab R2018b (https://it.mathworks.com/). Group comparison between clinical and radiological features of the two sites was performed using a χ-square to test sex, MS phenotype and disability progression, while a Mann–Whitney non parametric test was used for the remaining variables (age, disease duration, EDSS at baseline, time between visits, T2LL, GM, WM, thalamic and cerebellar volumes, as well as FA- and MD-NAWM values).

To avoid data redundancy and reduce variance in classifier performances, we investigated features’ co-linearity of clinical (age, sex, phenotype, disease duration, EDSS at baseline, time between visits and therapy at baseline) and neuroradiological (T2LL, GM, WM, thalamic and cerebellar volumes, FA- and MD-NAWM) features using a partial correlation analysis. Specifically, we performed partial correlation between each pair of features controlling for all the remaining features. For all analyses, a significance threshold was set for *p* < 0.01 without multiple comparison correction that allowed us to be conservative in the removal of confounding effects.

### ML classifiers

ML classifiers were performed and analyzed with Python, via the browser version of Jupyter Notebook application (https://jupyter.org/). We used a supervised ML technique and identified two binary classes: patients who were stable (negative output, 0) and patients who worsened in disability (positive output, 1) at the follow-up visit. We built a matrix whose rows represented patients and columns represented features. For each patient, features included clinical and neuroradiological data, MR acquisition site and class. A last column consisting of a randomly generated variable was added to the matrix. We applied a random forest algorithm for 1000 times and evaluated the confusion matrices of resulting classifiers.

In each of the 1000 classifiers, we used features calculated on samples from both sites to evaluate classifier performances. Patients from both sites were randomly selected to obtain the same numerosity in both classes (50% stable and 50% with disease progression patients) to avoid bias due to class numerosity. To train the ML classifier we randomly selected 80% of the sample with the same numerosity of stable and with disease progression patients and left the other 20% for validation. To avoid overfitting, we calculated each classifier accuracy both on the validation set and in a subsample of the training set and considered in the following analysis only classifiers showing differences in accuracies smaller than 0.02, i.e. Out-of-Bag test. The 0.02 threshold was selected being reasonably low to allow considering the results of the Out-of-Bag test and the training to be consistent, since there is no typical value reported in the literature, at the best of our knowledge.

Classifier performances were evaluated via accuracy, area under the true positive versus false positive rates curve (AUC), sensitivity (true positive rate) and specificity (true negative rate). Within each classifier including both clinical and neuroradiological features, permutation importance was calculated for each feature and for the random variable: only parameters whose importance was higher than the random variable’s importance, were considered actually relevant in the classifier.

## Results

### Sample selection

Average and standard deviation of clinical and neuroradiological features for the entire sample built from the two sites, as well as for both Site 1 and Site 2 separately, are reported in Table 1.

**Table 1:** Clinical and neuroradiological features

	All subjects (Site 1 + Site 2)	Subjects at Site 1	Subjects at Site 2	Between sites comparison
	Average (std)	Average (std)	Average (std)	*z*- (*p*-value)
Number	163	105	58	*–*
Age [years]	39.66 (10.23)	38.29 (9.75)	42.13 (10.68)	**− 2.43 (0.02)**
Sex (F/M)	104/59	80/25	24/34	19.61 (0.001)*
Phenotype (RR/P)	122/41	85/20	37/21	5.84 (0.02)*
Disease duration [years]	9.90 (8.06)	8.27 (7.97)	12.87 (7.40)	**− 3.85 (0.001)**
EDSS at baseline	3.0 [0.0–7.5]**	2.0 [0.0–7.5]**	3.5 [2.0–7.5]**	**− 5.48 (0.001)**
Time to follow-up [years]	3.93 (0.95)	4.2 (0.93)	3.38 (0.72)	**6.56 (0.001)**
Therapy (1st line, 2nd line, none)	53, 65, 45	32, 31, 42	21, 34, 3	–
Disability progression (Yes/No)	58/105	36/69	22/36	0.22 (0.64)*
T2LL [ml]	9.02 (10.31)	6.77 (6.94)	13.26 (13.62)	**− 4.47 (0.001)**
GM Volume [ml]	719.69 (92.18)	737.38 (84.62)	687.662 (97.32)	**3.48 (0.001)**
WM Volume [ml]	733.98 (96.91)	768.98 (84.31)	670.638 (85.94)	**6.14 (0.001)**
Thalamic Volume [ml]	17.64 (3.21)	18.04 (3.22)	16.92 (3.10)	**2.54 (0.001)**
Cerebellar Volume [ml]	113.66 (13.88)	110.016 (12.92)	120.25 (13.22)	**− 4.28 (0.01)**
FA-NAWM	0.40 (0.06)	0.43 (0.03)	0.33 (0.3)	**10.45 (0.001)**
MD-NAWM × 10^−3^	0.75 (0.05)	0.73 (0.07)	0.80 (0.04)	**− 8.68 (0.001)**

Clinical and neuroradiological features of Site 1 and Site 2 samples. Mann–Whitney test was used to test significant differences between groups. Significant differences are highlighted in bold font

*Std* standard deviation, *F* female; *M* male; *RR* relapsing remitting form; *P* progressive form, *EDSS* expanded disability status scale, *T2LL* T2 lesion load, *GM* Gray Matter, *WM* White Matter, *FA-NAWM* fractional anisotropy of normal appearing white matter, *MD-NAWM* mean diffusivity of normal appearing white matter

*χ-square statistics was used

**Median [range]

In the entire sample, participants were 39.66 ± 10.23 years old, range [19.50–70.30], with 104 females and 59 males. Of these 163 subjects, at baseline 122 patients had a relapsing–remitting form of MS, while the remaining 41 patients had a progressive form. Patients showed a disease duration of 9.90 ± 8.06 years, range [0.00–37.00], with a time between visits that was 3.93 ± 0.95 years, range [2–6]. At the follow-up examination, disease progression was observed in 58 over 163 patients (35.6% of the sample), whose EDSS distributions at baseline and at follow-up were respectively 3.5 [0.0–7.0] and 4.5 [1.5–7.0], while in the remaining 105 patients the EDSS remained stable at 3.0 [0.0–7.5].

Since patients who remained stable at follow-up were about 2/3 of both samples, and we aimed at balancing size of classes, at each of the 1000 performed models we picked all the patients who experienced disability progression from both sites’ sample and also randomly picked an equal number of patients who remained stable, reaching a final number of 72 participants from Site 1 and 44 participants from Site 2.

### Feature selection

Due to differences in demographic and clinical characteristics of participants between the two sites, we performed correlation analysis in the two samples separately. We found a number of features, which were inter-correlated, in both Site 1 and Site 2 samples (Tables 2, 3). After removing inter-correlated features (disease duration, MD-NAWM values, GM and WM volumes), ML classifiers included age, sex, disease phenotype, EDSS score and therapy at baseline, time between visits as clinical features, while T2LL, thalamic and cerebellar volumes and FA-NAWM were included as neuroradiological features.

**Table 2:** Correlation matrix, Site 1 sample

	AGE	DD	EDSS@base	T2LL	FA-NAWM	MD-NAWM	Thalamic volume	GM volume	WM volume	Cerebellar volume
AGE	–	**0.36 (0.003)**	0.18	− 0.02	− 0.12	− 0.25	0.01	− 0.14	0.08	− 0.18
DD		–	**0.32 (0.012)**	0.27	0.10	0.22	− 0.10	0.07	0.03	− 0.12
EDSS@base			–	0.11	0.15	0.08	0.07	0.02	− 0.19	− 0.16
T2LL				–	− 0.25	0.04	− 0.26	− 0.15	0.19	0.18
FA-NAWM					–	− **0.76 (0.001)**	− 0.01	− 0.20	0.13	0.29
MD-NAWM						–	− 0.11	− 0.05	0.06	0.15
Thalamic volume							–	0.28	**0.51 (0.001)**	− 0.05
GM volume								–	**0.36 (0.004)**	− 0.02
WM volume									–	0.14
Cerebellar volume										–

Multiple correlation analysis of clinical and radiological features. Spearman correlation coefficients are reported, significant correlations are highlighted in bold font and relative *p*-values are shown between round brackets

*DD* disease duration, *EDSS* expanded disability status scale, *T2LL* T2 lesion load, *FA-NAWM* fractional anisotropy of normal appearing white matter, *MD-NAWM* mean diffusivity of normal appearing white matter

**Table 3:** Correlation matrix, Site 2 sample

	AGE	DD	EDSS@base	T2LL	FA-NAWM	MD-NAWM	Thalamic volume	GM volume	WM volume	Cerebellar volume
AGE	–	**0.56 (0.001)**	− 0.07	− 0.14	0.10	0.00	0.03	− 0.11	0.04	− 0.31
DD		–	0.13	0.26	− 0.09	0.02	0.05	− 0.21	0.14	0.11
EDSS @base			–	0.23	0.17	0.23	0.13	− 0.13	0.00	− 0.23
T2LL				–	− 0.17	− 0.02	− 0.42	0.22	0.19	− 0.10
FA- NAWM					–	− **0.75 (0.001)**	0.30	− 0.01	− 0.14	0.16
MD- NAWM						–	0.20	0.01	− 0.10	− 0.06
Thalamic Volume							–	**0.57 (0.001)**	0.23	0.09
GM Volume								–	**0.56 (0.001)**	− 0.14
WM Volume									–	− 0.01
Cerebellar Volume										–

Multiple correlation analysis of clinical and radiological features. Spearman correlation coefficients are reported, significant correlations are highlighted in bold font and relative *p*-values are shown between round brackets

*DD* disease duration, *EDSS* expanded disability status scale, *T2LL* T2 lesion load, *FA-NAWM* fractional anisotropy of normal appearing white matter, *MD-NAWM* mean diffusivity of normal appearing white matter

### ML classifiers

**Fig. 2:**
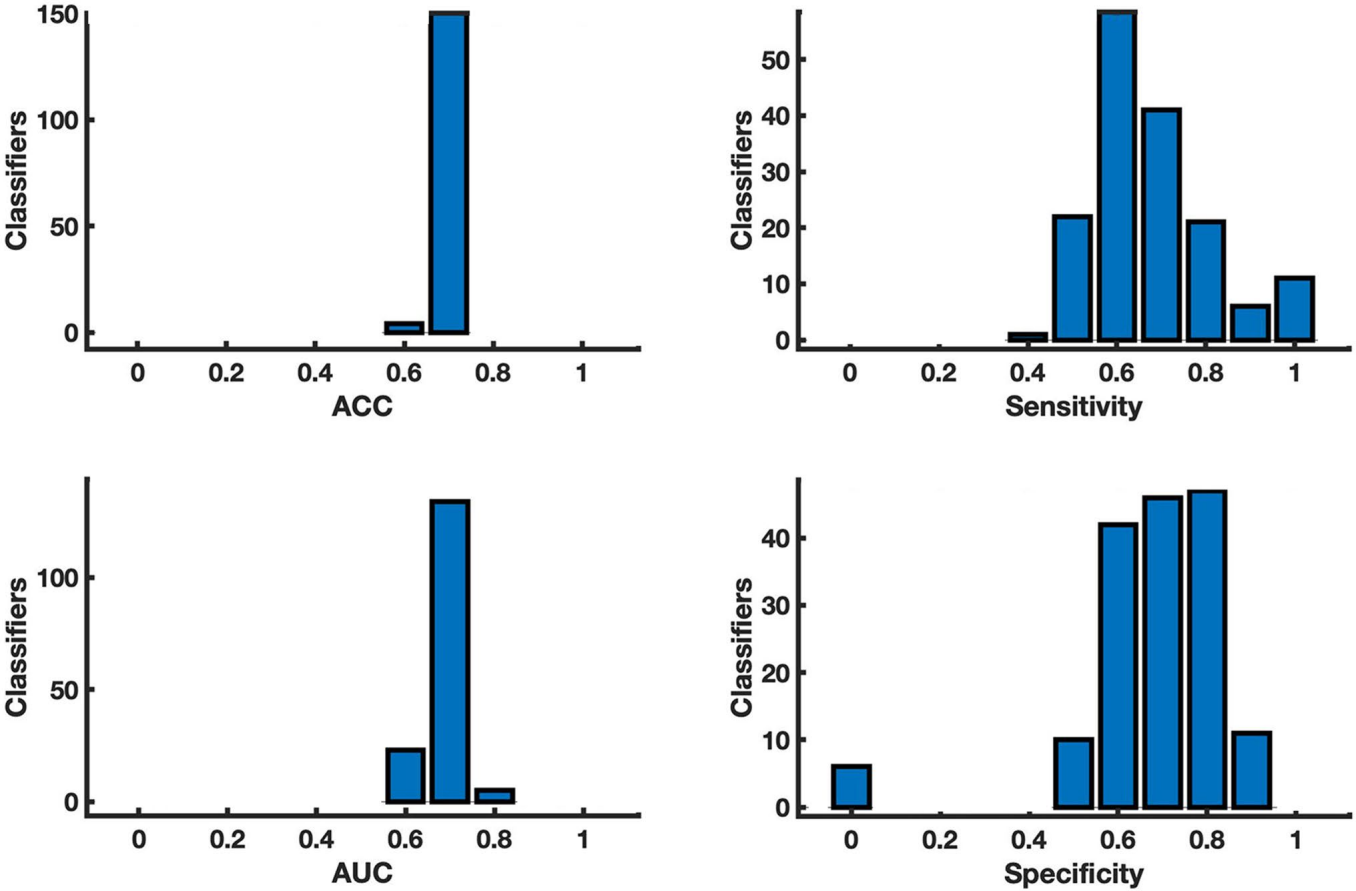
Metrics of classifier built on clinical and radiological features. Histogram of accuracy (ACC) and area under the true positive versus true negative rate curve (AUC), sensitivity and specificity obtained from the 150 classifiers, surviving the Out-of-Bag test, performed on the sample features. On the *y*-axis number of classifiers is displayed

Out of 1000 classifiers built on both clinical and neuroradiological features, 162 classifiers had a difference between the accuracy calculated in the validation set and in the subsample of the training set smaller than 0.02. Accuracy, AUC, sensitivity and specificity on these 162 classifiers are displayed in Fig. 2. The classifier with the highest resulting metrics proved to have an accuracy = 0.79, an AUC = 0.81, with a sensitivity and a specificity of 0.90 and 0.71, respectively (Table 4).

**Table 4:** Metrics

	*N*	Accuracy	AUC	Sensitivity	Specificity
ALL	162	0.79	0.81	0.90	0.71
Radiological	329	0.92	0.92	0.92	0.91
Clinical	128	0.71	0.72	0.69	0.75

Accuracy, area under the true positive versus true negative curve (AUC), sensitivity and specificity of the best performing machine learning classifier built on all clinical and radiological features, and either on clinical or radiological features. *N* represents the number of classifiers surviving the Out-of-Bag test

**Fig. 3:**
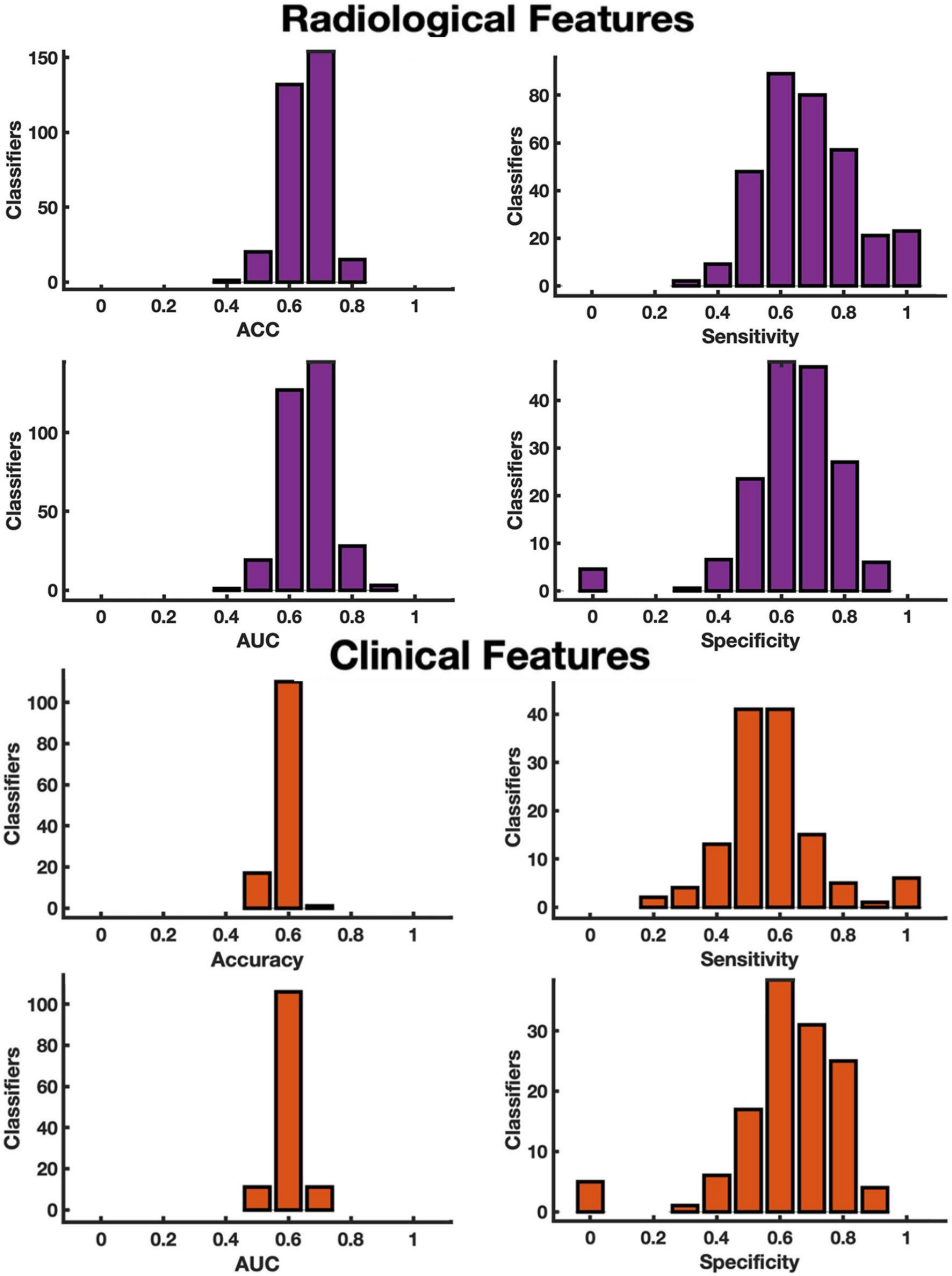
Metrics of classifier built on radiological/clinical features. Histograms of accuracy (ACC) and area under the true positive versus true negative rate curve (AUC), sensitivity and specificity, surviving the Out-of-Bag test obtained from the 309 classifiers built on neuroradiological features (top) and the 128 classifiers built on clinical features (bottom). On the y-axis number of classifiers is displayed

Then we tested separately classifiers built on neuroradiological features alone (T2LL, FA-NAWM, thalamic and cerebellar volume) and clinical features alone (age, sex, disease phenotype, EDSS score and therapy at baseline, time between visits). For the latter separated analyses, we followed the same procedure implemented to analyze neuroradiological and clinical features together, i.e. balancing the numerosity of class 0 and class 1, adding a random feature and validating for overfitting via the Out-of-Bag test. We found that classifiers built on neuroradiological features were more accurate and sensitive than those built on clinical features or on mixed clinical-neuroradiological features, showing better accuracy, AUC, sensitivity and specificity values (Table 4; Fig. 3).

**Fig. 4:**
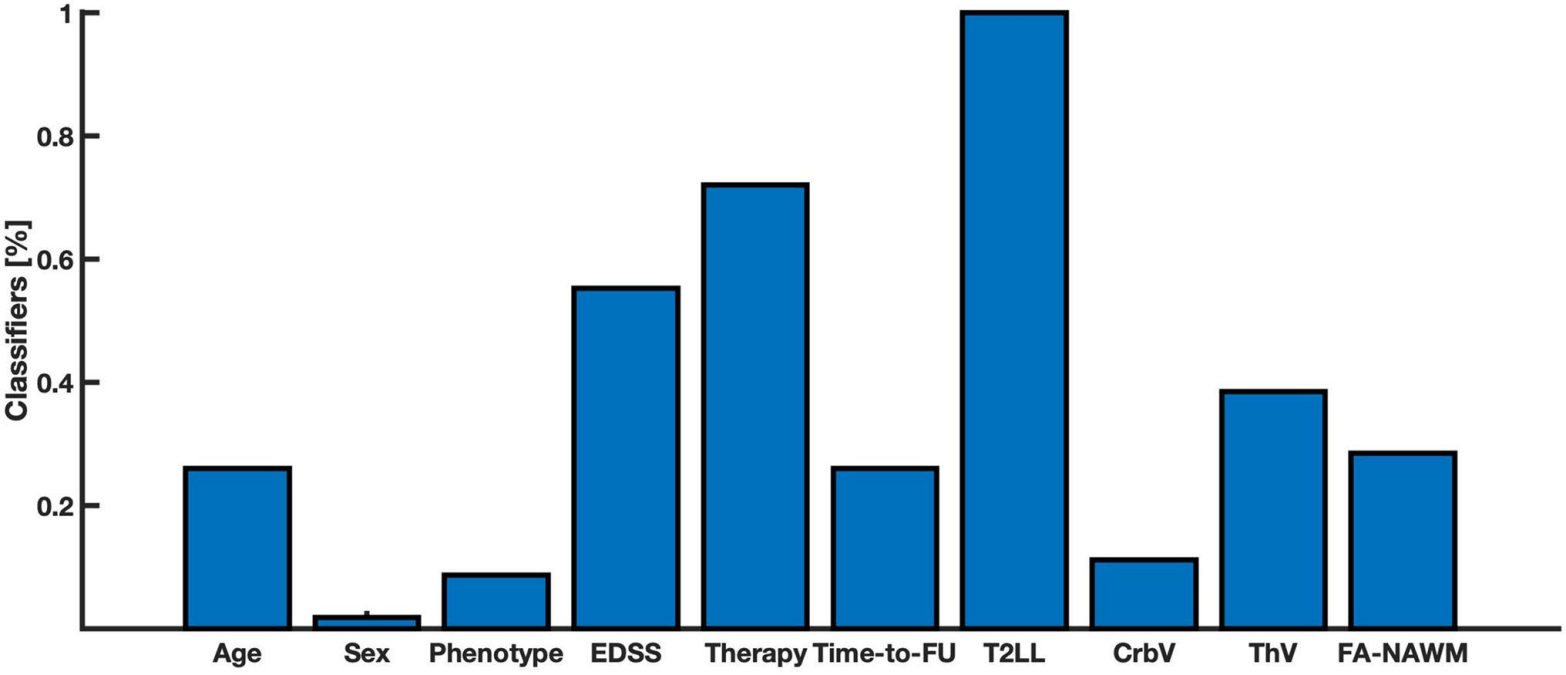
Important features frequency. Figure showing, for each feature, the percentage of time (i.e. classifiers) more important than the random feature in predicting disability progression in our MS group. *EDSSbase* expanded disability status scale at baseline, *TimeFU* time between first visit and follow-up, *CrbV* cerebellar gray matter volume, *GMV* gray matter volume, *FA-NAWM* fractional anisotropy of normal appearing white matter

To have a better depiction of features’ role in predicting disability progression, we further investigated the importance of each feature within each classifier built on both clinical and radiological features. T2LL was recognized as important feature in predicting disability progression in all the performed classifiers. EDSS and therapy at baseline were important in the 55% and 72% of the classifiers. Thalamic volume and FA-NAWM were important in 39% and 29% of the classifiers respectively. All the other radiological and clinical features were found to be more important than the random feature in a negligible number of classifiers (less than 10%, see Fig. 4).

## Discussion

We showed that MRI measures alone may predict disability progression with the highest accuracy, even in respect to the combination of neuroradiological and clinical features. We are prone to explain this last result as outsourcing from the risk of overfitting. Indeed, the risk of overfitting was higher in the classifiers including both clinical and neuroradiological features due to the numbers of features and subjects and allowed fewer classifiers to survive in respect to the original amount.

Even if we expect that combining both clinical and MRI data may predict clinical outcome at best [24], the role of neuroradiological features seems to be of primary relevance suggesting a more informative independent charge in respect to clinical features alone.

To strengthen the generalizability of the models obtained by the ML classifiers [25], we included in the study features characterizing subjects recruited in two sites, visited by four different clinicians and whose MRI were acquired with two different MR systems. As well, the observed subject samples covered wide ranges of age and disease severity. Therefore, our results may be generalizable and not site-specific, despite recruitment and acquisition protocols from the two sites may have produced differences in feature estimation. Further, we approached our research question with a supervised method, i.e. implementing classification algorithms, and it prevented us to perform less sophisticated methods like principal component analysis.

As Lew et al. [26] showed, ML algorithms may predict 2-year disability worsening in subjects with progressive disease. Our results extend the previous data, confirming the utility of a ML approach also for subjects in an earlier stage of the disease or with a lower level of disability. This is particularly relevant in this population of MS patients when clinicians have the opportunity to choose among several pharmacological treatments with different mechanism of actions and efficacy outcomes. Indeed, to identify disease features to drive neurologist in their choices is still a matter of debate across literature, given the absence of definitive shared prognostic factors of treatment choose and response [27].

Although we reached 79% of diagnostic accuracy, our results showed that our model was unable to provide the excellence [28]. Different reasons could explain this finding, ranging from a relatively small sample size to the not-linear relation between structural damage and disability, as it is quantified by the EDSS [29]. Nevertheless, it has to be considered that a sample size of 116 subjects may be enough to model disability progression from clinical and radiological features, as previously found [23, 30, 31]. Furthermore, our analysis included only parameters of structural brain damage, while the association between clinical findings and radiological extent of involvement is generally poor and known, given the clinico-radiological paradox in MS [32].

With regards to MRI data, our results indirectly confirm the importance of T2LL in predicting disease worsening, a variable known to influence both the disease course [24] and its progression [14, 33]. In particular, following the first episode of the disease, T2LL is helpful to identify those patients at risk of developing an aggressive form of MS [34]. This underlines how our result can help physicians in selecting those patients that can benefit from an early and more efficacy therapy. A recent study by Bakshi and colleagues found that baseline brain parenchymal fraction, but not T2LL, may be a good predictor of 5-year disability worsening [35]. A possible explanation to this discrepancy could be researched in the different clinical endpoint. In particular, authors considered longitudinal difference in the EDSS score as a disability marker, while we considered disability progression as defined in Rìo et al. [22]. Further, our results confirm the prognostic role of thalamus, whose measurement is reliable and comparable among centers, in MS [36]. Indeed, thalamic involvement in MS has been widely investigated using different approaches, with thalamic atrophy and function associated with poorer motor and cognitive performances [37, 38]. A recent and extensive work in 1417 subjects with MS identified key regions with early atrophy such thalamus consistently across MS phenotypes, exploring the sequence of GM atrophy and revealing the potentiality of therapeutic strategy adjustment based on the staging of patients [10, 39]. Finally, FA-NAWM values also proved to be a significant contributor to clinical progression in MS, in line with the established knowledge about the association between axonal loss and clinical deficit in MS [40], as well as that aberrant diffusivity measures in the NAWM may provide information in the relapsing remitting form of MS [41].

Lastly, in our analysis, both disability and therapy at baseline emerged as clinical features relevant to disability progression. In particular, therapy response is individual and depends on many factors, including age, phenotype and disability at treatment start [42], and is known to influence both disease course [43] and disability accumulation [44].

## Limitations

As mentioned before, a limitation of our study relies in the relatively small sample size, which might have affected the performance of ML classifiers in the prediction of MS disability, limiting their diagnostic accuracy. The small sample size also prevented us from using an independent test set to report final results [25]. Further, we used EDSS as index of disability. Even if depending mainly on walking ability, EDSS is widely used as disability measure [45]. More in general, the major limitation of this study has to be researched in its retrospective nature. For this reason, future prospective studies, properly designed to evaluate with additional techniques (e.g. fMRI, MTR, qMRI) other aspects of brain involvement, as well as other CNS structures (e.g. spinal cord) are strongly warranted, to verify our model and possibly increase its diagnostic accuracy.

## Conclusion

ML classifiers built on clinical and neuroradiological features may predict disability progression in subjects with MS at individual level with accuracy almost reaching a value of 80%. Among the MRI variables, T2LL and thalamic volume were the most important features in the prediction of disability progression, while disability and therapy at baseline were the most relevant features among the clinical variables. The implementation of classifiers based on neuroradiological and clinical features may aid clinicians to predict if a single subject may be prone to disability progression and consequently to tailor the treatment.

## Supplementary Information

Below is the link to the electronic supplementary material.

Supplementary file1 (DOCX 62 kb)

## Abbreviations

MS: Multiple sclerosis
ML: Machine learning
EDSS: Expanded disability status scale
T2LL: T2 lesion load
GM: Gray matter
CIS: Clinically isolated syndrome
WM: White matter
FA-NAWM: Fractional anisotropy of normal appearing white matter
MD-NAWM: Mean diffusivity of normal appearing white matter
AUC: Area under the curve

## References

[1] Confavreux C, Vukusic S (2014). The clinical course of multiple sclerosis. Handb Clin Neurol.

[2] Ciccarelli O, Barkhof F, Bodini B (2014). Pathogenesis of multiple sclerosis: insights from molecular and metabolic imaging. Lancet Neurol.

[3] Amato MP, Fonderico M, Portaccio E (2020). Disease-modifying drugs can reduce disability progression in relapsing multiple sclerosis. Brain.

[4] Tommasin S, Giannì C, De Giglio L, Pantano P (2017). Neuroimaging techniques to assess inflammation in multiple sclerosis. Neuroscience.

[5] Tintore M, Rovira À, Río J (2015). Defining high, medium and low impact prognostic factors for developing multiple sclerosis. Brain.

[6] Pontillo G, Cocozza S, Di Stasi M (2020). 2D linear measures of ventricular enlargement may be relevant markers of brain atrophy and long-term disability progression in multiple sclerosis. Eur Radiol.

[7] Radue E-W, Barkhof F, Kappos L (2015). Correlation between brain volume loss and clinical and MRI outcomes in multiple sclerosis. Neurology.

[8] Louapre C, Bodini B, Lubetzki C (2017). Imaging markers of multiple sclerosis prognosis. Curr Opin Neurol.

[9] Filippi M, Brück W, Chard D (2019). Association between pathological and MRI findings in multiple sclerosis. Lancet Neurol.

[10] Eshaghi A, Prados F, Brownlee WJ (2018). Deep gray matter volume loss drives disability worsening in multiple sclerosis. Ann Neurol.

[11] Cocozza S, Petracca M, Mormina E (2017). Cerebellar lobule atrophy and disability in progressive MS. J Neurol Neurosurg Psychiatry.

[12] D’Ambrosio A, Pagani E, Riccitelli GC (2017). Cerebellar contribution to motor and cognitive performance in multiple sclerosis: an MRI sub-regional volumetric analysis. Mult Scler.

[13] Patti F, De Stefano M, Lavorgna L (2015). Lesion load may predict long-term cognitive dysfunction in multiple sclerosis patients. PLoS ONE.

[14] Calabrese M, Poretto V, Favaretto A (2012). Cortical lesion load associates with progression of disability in multiple sclerosis. Brain.

[15] Wilkins A (2017). Cerebellar dysfunction in multiple sclerosis. Front Neurol.

[16] Rocca MA, Mesaros S, Pagani E (2010). Thalamic damage and long-term progression of disability in multiple sclerosis. Radiology.

[17] Datta G, Colasanti A, Rabiner EA (2017). Neuroinflammation and its relationship to changes in brain volume and white matter lesions in multiple sclerosis. Brain.

[18] Wottschel V, Alexander DC, Kwok PP (2015). Predicting outcome in clinically isolated syndrome using machine learning. Neuroimage Clin.

[19] Zurita M, Montalba C, Labbé T (2018). Characterization of relapsing-remitting multiple sclerosis patients using support vector machine classifications of functional and diffusion MRI data. Neuroimage Clin.

[20] Polman CH, Reingold SC, Banwell B (2011). Diagnostic criteria for multiple sclerosis: 2010 revisions to the McDonald criteria. Ann Neurol.

[21] Thompson AJ, Banwell BL, Barkhof F (2018). Diagnosis of multiple sclerosis: 2017 revisions of the McDonald criteria. Lancet Neurol.

[22] Río J, Nos C, Tintoré M (2006). Defining the response to interferon-beta in relapsing-remitting multiple sclerosis patients. Ann Neurol.

[23] Raz E, Cercignani M, Sbardella E (2009). Clinically isolated syndrome suggestive of multiple sclerosis: voxelwise regional investigation of White and Gray matter. Radiology.

[24] Zhao Y, Healy BC, Rotstein D (2017). Exploration of machine learning techniques in predicting multiple sclerosis disease course. PLoS ONE.

[25] Bluemke DA, Moy L, Bredella MA (2020). Assessing radiology research on artificial intelligence: a brief guide for authors, reviewers, and readers—from the radiology editorial board. Radiology.

[26] Law MT, Traboulsee AL, Li DK (2019). Machine learning in secondary progressive multiple sclerosis: an improved predictive model for short-term disability progression. Mult Scler J Exp Transl Clin.

[27] Gasperini C, Prosperini L, Tintoré M (2019). Unraveling treatment response in multiple sclerosis: a clinical and MRI challenge. Neurology.

[28] Šimundić A-M (2009). Measures of diagnostic accuracy: basic definitions. EJIFCC.

[29] Schoonheim MM, Geurts JJG, Barkhof F (2010). The limits of functional reorganization in multiple sclerosis. Neurology.

[30] Calabrese M, Mattisi I, Rinaldi F (2010). Magnetic resonance evidence of cerebellar cortical pathology in multiple sclerosis. J Neurol Neurosurg Psychiatry.

[31] Davie CA, Barker GJ, Webb S (1995). Persistent functional deficit in multiple sclerosis and autosomal dominant cerebellar ataxia is associated with axon loss. Brain.

[32] Barkhof F (2002). The clinico-radiological paradox in multiple sclerosis revisited. Curr Opin Neurol.

[33] Elliott C, Belachew S, Wolinsky JS (2019). Chronic white matter lesion activity predicts clinical progression in primary progressive multiple sclerosis. Brain.

[34] Tintore M, Arrambide G, Otero-Romero S (2019). The long-term outcomes of CIS patients in the Barcelona inception cohort: Looking back to recognize aggressive MS. Mult Scler.

[35] Bakshi R, Healy BC, Dupuy SL (2020). Brain MRI predicts worsening multiple sclerosis disability over 5 years in the SUMMIT study. J Neuroimaging.

[36] Dwyer M, Brior D, Lyman C (2020). Artificial intelligence-based thalamic volumetry is fast, reliable, and generalizable to large, heterogeneous datasets using only clinical quality T2-FLAIR MRI (4846). Neurology.

[37] Azevedo CJ, Cen SY, Khadka S (2018). Thalamic atrophy in multiple sclerosis: a magnetic resonance imaging marker of neurodegeneration throughout disease. Ann Neurol.

[38] Tona F, Petsas N, Sbardella E (2014). Multiple sclerosis: altered thalamic resting-state functional connectivity and its effect on cognitive function. Radiology.

[39] Stankoff B, Louapre C (2018). Can we use regional grey matter atrophy sequence to stage neurodegeneration in multiple sclerosis?. Brain.

[40] Haines JD, Inglese M, Casaccia P (2011). axonal damage in multiple sclerosis. Mt Sinai J Med.

[41] Kolasa M, Hakulinen U, Brander A (2019). Diffusion tensor imaging and disability progression in multiple sclerosis: a 4-year follow-up study. Brain Behav.

[42] Kalincik T, Manouchehrinia A, Sobisek L (2017). Towards personalized therapy for multiple sclerosis: prediction of individual treatment response. Brain.

[43] Cree BAC, Mares J, Hartung H-P (2019). Current therapeutic landscape in multiple sclerosis: an evolving treatment paradigm. Curr Opin Neurol.

[44] Hart FM, Bainbridge J (2016). Current and emerging treatment of multiple sclerosis. Am J Manag Care.

[45] Cohen JA, Reingold SC, Polman CH (2012). Disability outcome measures in multiple sclerosis clinical trials: current status and future prospects. Lancet Neurol.

