# Supplementary material for: Machine learning classifier to identify clinical and radiological features relevant to disability progression in multiple sclerosis
Source: J Neurol. 2021 May 10;268(12):4834–45. doi: 10.1007/s00415-021-10605-7 (PMC8563671; doi:10.1007/s00415-021-10605-7)
Supplement: Supplementary file 1 — Supplementary file1 (DOCX 62 kb) [file 415_2021_10605_MOESM1_ESM.docx]

**Supplementary Material.**

**Table S1. MRI protocols.**

|  | **Site 1** | **Site 2** |
| --- | --- | --- |
| **T13D** | *MPRAGE* | *MPRAGE* |
| - *Orientation* | Sagittal | Sagittal |
| - *Number of Slices* | 176 | 176 |
| - *Voxel Size [mm^3^]* | 1x0.51x0.51 | 1x1x1 |
| - *TR [ms]* | 1900 | 2500 |
| *- TE [ms]* | 2.93 | 2.8 |
| - *TI [ms]* | 900 | 900 |
| - *Flip angle* | 9° | 9° |
| - *Matrix* | 256x256 | 256x256 |
| - *FOV [mm^2^]* | 260 | 256 |
| **T2** | *Dual Echo* | *FLAIR* |
| - *Orientation* | Sagittal | Sagittal |
| - *Number of Slice* | 25 | 176 |
| - *Voxel Size [mm^3^]* | 0.57x0.57x4 | 1x1x1 |
| - *TR [ms]* | 3320 | 6000 |
| - *TE [ms]* | 10/103 | 396 |
| - *TI [ms]* | - | 2200 |
| - *Flip angle* | 150° | 120° |
| - *Matrix* | 384x384 | 256x256 |
| - *FOV [mm^2^]* | 220 | 256 |
| - *gap* | 30% | 0 |
| **DWI** | *Echo-planar* | *Echo-planar* |
| - *Orientation* | Axial | Axial |
| - *Number of Slice* | 72 | 45 |
| - *Voxel Size [mm^3^]* | 2x2x2 | 1.8x1.8x3.3 |
| - *TR [ms]* | 12200 | 5200 |
| - *TE [ms]* | 94 | 82 |
| - *Matrix* | 96 × 96 | 128x128 |
| - *FOV [mm^2^]* | 192 | 230 |
| - *N Directions* | 30 | 64 |
| - *b-factors* | 0-1000 s/mm^2^ | 0-1000 s/mm^2^ |
